# Dodecyl creatine ester therapy: from promise to reality

**DOI:** 10.1007/s00018-024-05218-y

**Published:** 2024-04-17

**Authors:** Aloïse Mabondzo, Jiddeke van de Kamp, Saadet Mercimek-Andrews

**Affiliations:** 1 Paris Saclay University, CEA, Medicines and Healthcare Technologies Department (MTS), SPI, Neurovascular Unit Research and Therapeutic Innovation Laboratory, 91191 Gif-sur-Yvette cedex, France; 2 Department of Human Genetics, Amsterdam UMC, Vrije Universtiteit Amsterdam, Amsterdam, The Netherlands; 3 Department of Medical Genetics, Faculty of Medicine and Dentistry, Neurosciences and Mental Health Institute, University of Alberta, Edmonton, AB Canada

**Keywords:** Creatine transporter deficiency, Nose-to-brain drug delivery, Clinical trial, Biomarkers, Blood–brain barrier, Cognitive functions, Proton MRS

## Abstract

Pathogenic variants in *SLC6A8*, the gene which encodes creatine transporter SLC6A8, prevent creatine uptake in the brain and result in a variable degree of intellectual disability, behavioral disorders (e.g., autism spectrum disorder), epilepsy, and severe speech and language delay. There are no treatments to improve neurodevelopmental outcomes for creatine transporter deficiency (CTD). In this spotlight, we summarize recent advances in innovative molecules to treat CTD, with a focus on dodecyl creatine ester, the most promising drug candidate.

Creatine transporter deficiency (CTD) is a disorder caused by pathogenic variants (homozygous in males and heterozygous in females) in *SLC6A8*, leading to lack of creatine in the brain and especially in neurons. Its primary neurological phenotypes are intellectual disability, behavioral disorders (e.g., autism spectrum disorder), epilepsy (usually responsive to anti-epileptic drugs), and severe speech and language delay [18]. CTD patients also often (about of 60% patients) present locomotor activity disorders or dysfunction.

Brain magnetic resonance spectroscopy (MRS) shows near absence of creatine in the whole brain [18]. As an X-linked disorder, CTD affects males more severely and causes about 1–2% of all X-linked intellectual disability [14]. Consistent with variable X-inactivation, females can be asymptomatic but can also present with similar features as male patients [12, 19]. There are no treatments for CTD approved by the US Food and Drug Administration (FDA) or European Medicines Agency, leaving patients and caregivers with no option but supportive care [5]. The severity of CTD symptoms results in strong unmet medical needs and thus it is imperative to develop novel treatments to improve neurodevelopmental outcomes.

Creatine has an important role in energy metabolism. At the sites of energy production, newly produced ATP is used to phosphorylate creatine in a creatine kinase-mediated reaction. The resulting ADP is available to generate ATP. The resulting phosphocreatine diffuses into the cytosol. At the sites of energy utilization, phosphocreatine donates its phosphate to ADP to generate ATP, again in a creatine kinase-mediated reaction. The creatine-phosphocreatine shuttle allows for greater energetic density in cells and regulates energetic pathways triggered by ADP:ATP ratios.

Treating CTD appears simple: transport creatine into the brain cells. In individuals with creatine synthesis disorders, increasing brain creatine levels thanks to oral supplementation of creatine monohydrate and/or precursors should improve neurodevelopmental outcomes. This task has proven more daunting than expected in CTD since oral creatine supplementation or arginine and glycine precursors do not increase brain creatine concentrations [4, 18], despite seizure cessation and modest cognitive improvement in some patients [1, 8]. This creates a need for strategies enabling creatine to enter the brain in sufficient quantity to reach the target cells, and restore the endogenous function of creatine. Such a therapeutic strategy would need to be lifelong and to start early in life, making long-term safety investigations essential.

The most straightforward strategy for restoring creatine levels in brain cells is first to circumvent the blood–brain barrier (BBB) and second to ensure entry into the neurons regardless of creatine transporter activity. This primarily involved the repositioning of old molecules structurally related to creatine for passive diffusion across the BBB. An attempt was made to use cyclocreatine, which masks the positive charge on the guanidino terminal of creatine and has a planar structure. It was shown that cyclocreatine entered the brain of *Slc6a8* knockout (*Slc6a8*^*−/y*^) mice and improved the cognitive deficits seen in these mice with low efficacy. However, concerns regarding the toxicity of cyclocreatine in rats and beagles caused the suspension of human clinical trials [6, 20].

Intranasal delivery of creatine has been investigated in an animal model of stroke [3]. The authors demonstrated the reduction of infarction in *Slc6a8*^*−/y*^ mice. The proton–HR-MAS NMR analysis showed that intranasal delivery of creatine elevated the creatine/phosphocreatine peaks and the creatine/*N*-acetylaspartate ratio in the contralateral as well as the ipsilateral cerebral cortex of *Slc6a8*^*−/y*^ mice. The elevated creatine/phosphocreatine peaks indicate the presence of extratissular creatine, but not the presence of creatine in brain neurons.

Instead of altering the molecular structure of creatine, another strategy is to develop prodrugs that mask the polar ends, allowing passive diffusion through biological membranes. Dodecyl creatine ester (DCE) is a prodrug of creatine selected from a library of creatine prodrugs that link the hydroxyl group of creatine with a dodecyl alcohol, making the lipophilic ester [15]. When cultured fibroblasts from CTD patients were incubated with DCE, there was an increase in creatine concentration, showing that DCE is converted to creatine within the cells [16]. Both intranasal and intracerebroventricular administration of DCE increased brain creatine concentrations and improved cognitive function in *Slc6a8*^*−/y*^ mice [17]. In a subsequent study, mice were treated for 14 days prior to and through testing, for a total of 30 days. Extended DCE treatment improved short- and long-term recognition memory and spontaneous alternation rate in novel object recognition tests in a different *Slc6a8*^*−/y*^ mouse model [9]. Such efforts need to be pursued in the evaluation of other cognitive behaviors. Treatment with DCE increased the concentration of creatine in deep brain structures and especially neurons, confirming that a very small amount of DCE delivers creatine to the brain where it enters the creatine-phosphocreatine shuttle system.

These findings highlight that a very small dose of creatine will enter brain cells thanks to the nose-to-brain pathway, which bypasses the BBB and provides direct access to the brain. It seems to be sufficient to improve cognitive function. Little is known about the underlying mechanisms of creatine-mediated behavioral disorders. Correlating proteomic changes with behavioral disorders could provide mechanistic insights into creatine-mediated changes. Functional imaging and neuronal activation studies show that sub-brain regions such as the cortex, cerebellum, and hippocampus are involved in the storage and retrieval and formation of spatial memory [11]. Our recently published data provide new evidence regarding the efficacy of DCE therapy in the restoration of cognitive function in CTD [9]. Shotgun proteomics analysis, integrative bioinformatics, and statistical modeling have identified 14 key proteins, including kinesin-like protein KIF1A, also known as axonal transporter of vesicle synaptic BDNF, and phospholipase C proteins (PLCB1) that are dysregulated in the cerebellum, cortex, hippocampus, and brain stem of *Slc6a8*-/y mice compared with wild-type mice and that are recovered by DCE. The abundance of these proteins in the four brain regions was significantly correlated with both the object recognition and the Y-maze tests. These results confirm what has been reported in the literature regarding the association of KIF1A and PLCB1 with cognitive function in different brain disorders [7, 10]. Our findings suggest a major role for PLCB1, KIF1A, and associated molecules in the pathogenesis of CTD. These findings suggest a profound alteration of the molecular landscape in the brain of *Slc6a8*^*−/y*^ mice that could be recovered with DCE treatment. This study highlights how a single protein transporter dysfunction can significantly alter brain biochemistry, potentially playing a crucial role in the intellectual disability in CTD patients. However, since studies of cyclocreatine have shown how a narrow therapeutic window can hamper the translational potential of treatments showing efficacy in animal models, translational studies are needed, such as the use of cultures of brain organoids and fluids from CTD patients, so as to investigate whether these biomarkers are changed in the plasma or cerebrospinal fluid of CTD patients, in order to validate the significance of the key signatures linked to cognitive dysfunction. In any case, these data significantly enhance our understanding of CTD, offering potential therapeutic targets and a robust foundation for continued research in the field.

A new pharmaceutical formulation is optimized to guarantee DCE stability over time. Since the treatment is lifelong, chronic, short- and long-term safety of intranasal DCE therapy should be assessed in human clinical trials to pave the way to FDA approval to bring this therapy to market. The first human intranasal administration of DCE will shed light on the potential to improve neurodevelopmental outcomes in CTD from the safety and efficacy point of view, as well as complying with regulatory expectations. However, monitoring target engagement and pharmacodynamic responses in CTD patients is a challenge. Any restoration of cognitive dysfunction in DCE-treated patients will likely take several weeks to months to manifest.

Therefore, we need relevant outcome measures for CTD patients (29) as well as biomarkers that show DCE treatment is engaging the target systems. Proton MRS can detect creatine in the whole brain, but has several limitations, especially a lack of detailed measurements of creatine within the brain cells, i.e., the site of action, or brain proteins involved in the pathophysiology of CTD, as well as high cost and the frequent need for general anesthesia in CTD patients. Therefore, better biomarkers are needed to monitor the efficacy of future treatments of CTD. Our recent findings [9, 17] highlight increases in creatine levels in the hippocampus, cortex, striatum, and cerebellum of DCE-treated Slc6a8-/y mice, an increase in brain BDNF expression [9], as well changes in alteration of energy metabolism using PET imaging as part of a 18F-DG test (Fig. 1). Increases in energy metabolism could be reflected by an increased ATP/ADP ratio and by high-energy purine and pyrimidine metabolites or modulation of other neurodegenerative biomarkers such as tau protein [13]. Identification of these peripheral biomarkers in relation to changes of markers associated with cognitive function and cerebral energy metabolism is required for clinical and research purposes.

**Fig. 1:**
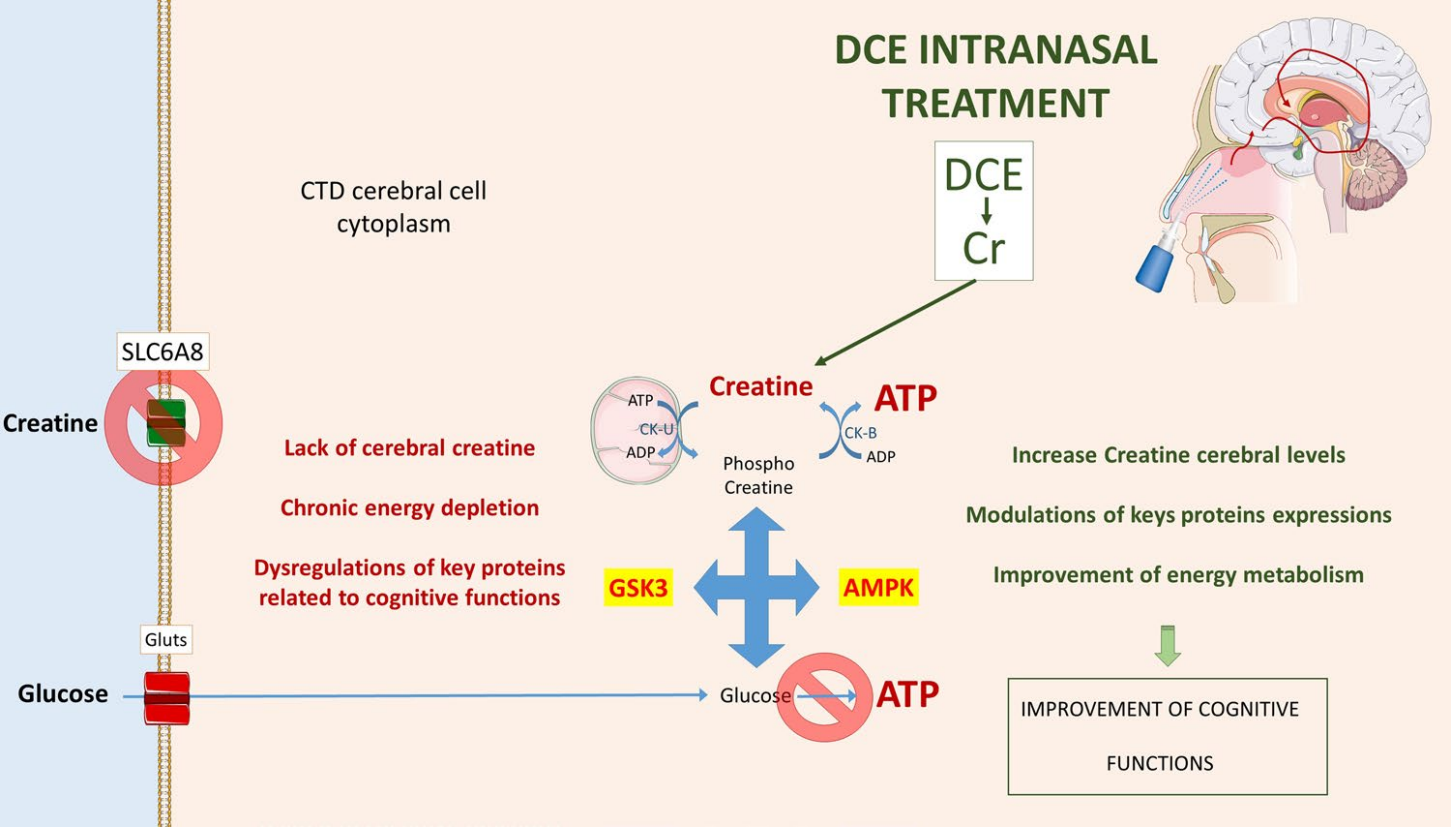
Schematic representation of the link between DCE intranasal treatment and the improvement of cognitive functions

The DCE-based therapeutic approach has the potential to improve the treatment of CTD patients as highlighted above, and since administration of DCE has to be lifelong, precise human toxicology data are required, and since clinical change may take months, research needs to continue to monitor urine and blood biomarkers.

## Data Availability

Not applicable.
